# Effects of mixed reality head-mounted glasses during 90 minutes of mental and manual tasks on cognitive and physiological functions

**DOI:** 10.7717/peerj.5847

**Published:** 2018-11-06

**Authors:** Carole Cometti, Christos Païzis, Audrey Casteleira, Guillaume Pons, Nicolas Babault

**Affiliations:** 1 Centre d’Expertise de la Performance, U1093 INSERM, Université de Bourgogne, Dijon, France; 2 Framatome, Lyon, France

**Keywords:** Balance, Mental, Electromyography, Precision, Autonomy, Heart rate, Fatigue

## Abstract

**Background:**

We evaluated the effects of a mixed reality (MR) head-mounted deviceon some cognitiveand physiological functions during 90 min tasks in an attempt to determine their safety for workers.

**Methods:**

A total of 12 volunteers performed 90-min intellectual and manual tasks with and without MR glasses. Balance, Stroop, and memory tests were conducted before, during and after these tasks. Heart rate and electromyographic activity of some muscles were recorded. A survey was used to determine subjective fatigue, pain, or discomfort.

**Results:**

Balance, heart rate, rate of perceived exertion, memory, and attention were unaffected by wearing MR glasses. Electromyographic activity increased with MR glasses for deltoid, biceps brachii, and soleus muscles. Few subjects reported discomfort, pain, or visual fatigue with MR glasses. Some participants reported they lost the notion of time and reality.

**Discussion:**

Accordingly, we concluded that the MR glasses under investigation (Hololens) can be used safely. An appropriate setup and familiarization are needed to optimize use.

## Introduction

Virtual reality (VR, a completely artificial environment accepted as real by users), augmented reality (AR, an integration of digital information in a user’s real environment) and mixed reality (MR, independent use of reality, VR or AR using immersive technologies) have become increasingly popular, with developments such as head-mounted displays, haptic devices, and glasses (Kim, Kim & Kim, 2017). VR/AR/MR have applications in research, education, rehabilitation, advertisement, and entertainment (Tarr & Warren, 2002; Bohil, Alicea & Biocca, 2011; Mirelman et al., 2016; Jouary et al., 2016; Watanabe et al., 2017; Kroes et al., 2017).

In medicine, AR technology is used to guide surgery interventions with more pertinent information. Results of recent studies demonstrated the effectiveness of AR glasses technology as an important adjunct to simulated medical skills training (Badiali et al., 2014; Rochlen, Levine & Tait, 2016) or during surgery (Tepper et al., 2017; Zhu et al., 2017). In the area of work and safety, several studies highlighted the benefits of AR. In one study examining scaffolding environments where workers undertook training before working with AR models of elevated constructions, authors found that workers increased their stride length with the repetitions of the task (Hsiao et al., 2005). This likely revealed increased confidence or habit-related learning effects. AR devices might improve balance-control, which in turn might reduce the risk of falls before workers even enter a construction job.

In contrast, some studies showed alterations in physiological functions with these devices. Ferrari et al. (2014) found increased oxygenation over the prefrontal cortex during a balance task performed in a semi-immersive VR environment (with increased difficulty). Other studies demonstrated that long exposure to VR/AR could provoke cyber sickness with a slowing of reaction time that was correlated with nausea, heart rate increases (Nalivaiko et al., 2015), gastric tachyarrhythmia, eye blink rate, and electroencephalographic delta waves (Kim et al., 2005). Some authors concluded that stomach activity, blinking, and breathing are good estimates for cyber sickness with VR devices (Dennison, Wisti & D’Zmura, 2016). However, a weak link has been found between postural instability and such cyber sickness (Dennison et al., 2017).

Screens, whatever the type, also induce visual fatigue. For instance, visual fatigue produced by smartphones have negative effects on balance function (Park, An & Moon, 2017). In order to avoid these effects, a correct posture and frequent rest periods are recommended (Kim & Koo, 2016). Concerning AR eyeglasses, Gamberini et al. (2015) evaluated users’ experience and demonstrated that the head-mounted device under investigation was comfortable regardless of the context of use. Overall, the experience of using the AR glasses was pleasant, but possible issues related to visual fatigue (Gamberini et al., 2015) or increased cognitive load (Baumeister et al., 2017) emerged. In a more recent study, authors showed participant tolerance using a commercially available headset without any effect on memory (Kroes et al., 2017). Further evaluation of this innovative technology under a variety of simulated conditions, for example, with a perspective for workers, would be an important issue.

To date, research related to this technology has received little attention. To our knowledge, no study has investigated the effects of MR glasses on motor control or on physiological and cognitive functions and more particularly during long-duration uses. Accordingly, the present study aimed to investigate the effects of Hololens MR glasses on some cognitive and physiological functions during and after different intellectual and manual tasks in an attempt to determine its safety. We tested the effects of MR glasses worn for 90 min with special interest in cardiovascular (heart rate), neuromuscular (electromyography (EMG) and balance) and cognitive (memory, fatigue, and attention) functions. In contrast to a purely virtual environment, and due to the fact that MR independently uses reality, VR, and AR, we hypothesized that MR would only slightly alter these measurements and more particularly subjective ratings (e.g., visual fatigue) and balance. If this hypothesis is upheld, we could conclude that MR devices can be safely used, particularly in working environments.

## Materials and Methods

### Participants

A total of 12 volunteers (five women and seven men) were recruited for this study (mean ± standard deviation: age 27.2 ± 5.3 years; height, 172.6 ± 7.4 cm; body mass 63.1 ± 16.1 kg). All participants were healthy with no injury within the preceding 6 months. Prior to participation, volunteers were fully informed about the purpose of the study and experimental procedure. All signed an informed consent form. The study was conducted according to the Declaration of Helsinki and approval was obtained from the committee on human research of the Sport Science faculty (CEP1701). During the duration of the study, subjects were instructed to refrain from intensive exercise.

### Experimental design

During this randomized, crossover, controlled study, participants went to the laboratory on three separate occasions (one familiarization and two experimental sessions) with a minimum of 3 days in-between. After inclusion, participants attended a familiarization session during which they received information about the main functions of the MR glasses, performed simple tasks to learn how to navigate within the system, familiarizing themselves with its functions, and run applications necessary for the experimental sessions. The familiarization session lasted at least 30 min, more when participants took longer to become familiar with the MR device.

**Figure 1:**
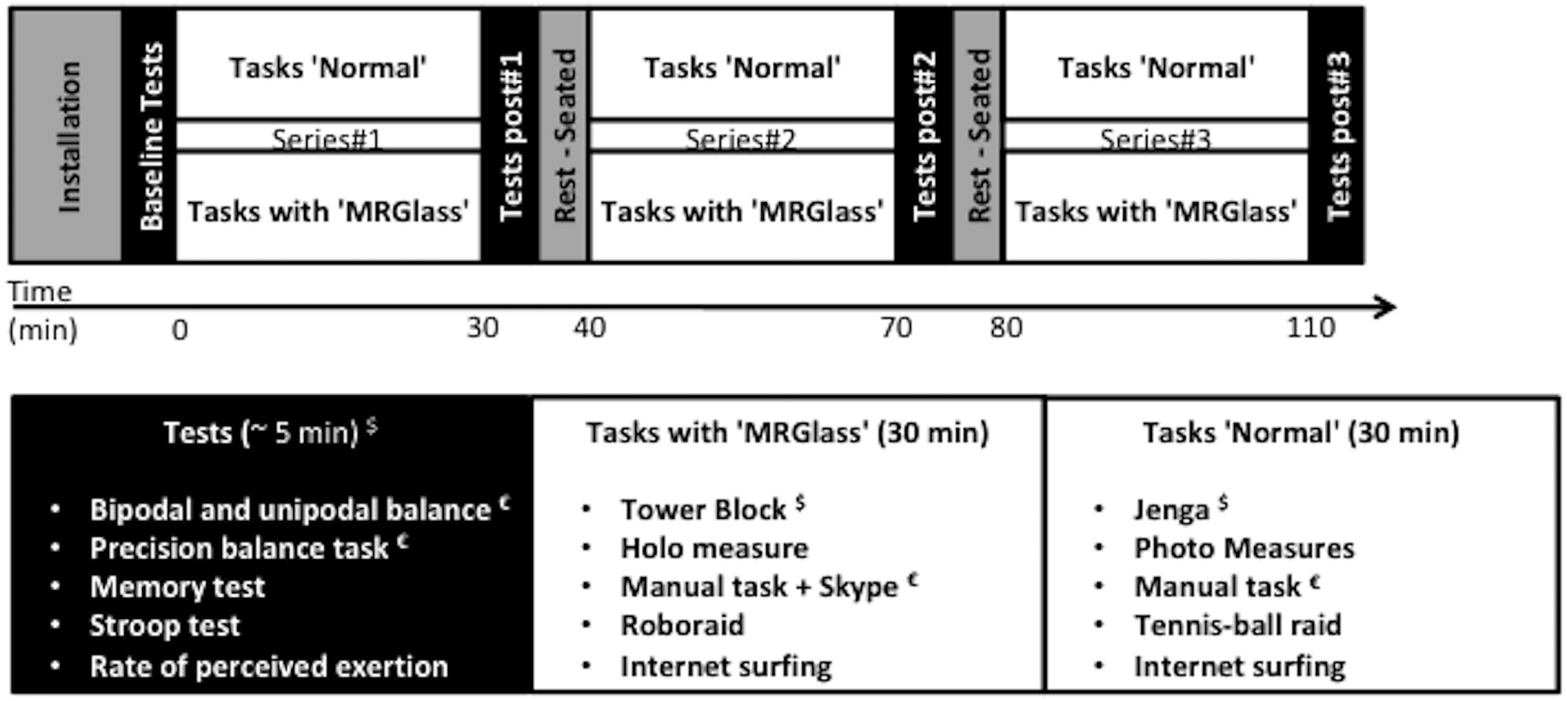
Experimental setup. $, heart rate measurements; €, electromyographic recordings.

The experimental sessions were performed under two conditions, wearing and not wearing MR glasses, designated hereafter as “MRGlass” and “Control,” respectively, in random order. The order for a given participant was obtained by drawing one of two pieces of papers from a black box. During each experimental session, participants performed three 30-min series of intellectual and manual tasks that included some games, a construction of a specific device and Internet surfing (see below). Accordingly, the total duration of the different tasks was 90 min. The different tasks were chosen in order to allow replication during both experimental conditions (MRGlass and Control). Moreover, during some tasks, participants were requested to move around inside the experimental room. Tests measuring cardiovascular, neuromuscular, and cognitive functions were made at baseline and after each series of tasks (post#1, post#2, post#3) (Fig. 1). At the beginning of each session, participants were equipped with a portable heart rate monitor and wireless electromyographic electrodes and received information about the session timeline. The baseline tests were then conducted, immediately followed by the first 30-min series of intellectual and manual tasks. The tests (baseline and post-task) lasted 10 min and were followed by a 10 min rest period in the sitting position before the next series of tasks.

In both conditions, participants were allowed to wear their corrective lenses if needed (*n* = 4).

### MRGlass condition

During the MRGlass session, participants were equipped with a commercially available head-mounted HoloLens MR device (Microsoft Corporation, Redmond, WA, USA). It was worn when performing the tasks and during the function tests, but was removed during the rest periods.

Participants were requested to perform five different tasks (each lasting 6 min) always presented in the same order using commercially available applications: (i) Tower block, (ii) Holo measure, (iii) Manual task associated with a Skype call, (iv) Roboraid, and (v) Internet surfing. Briefly, Tower block (Valorem Consulting Group LLC, Kansas city, MO, USA) consisted in a virtual tower made of wood blocks. Participants were instructed to remove blocks from an existing tower and place them one by one on the top of the tower so as to make it as tall as possible without making it fall. For Holo measure (Sebastians Apps powered by Unity technologies Inc., San francisco, CA, USA), participants were asked to measure different objects and the room size according to the experimenter’s instructions. The manual task consisted in the construction of a specific running speed system (Optojump, Microgate, Bolzano, Italy). The experimenter interacted with the participant as needed via Skype (Microsoft Corporation, Redmond, WA, USA) to help for the construction, and displaying the corresponding instruction manual on the screen. Roboraid (Microsoft Corporation, Redmond, WA, USA) was a game involving virtual laser shots made by robots coming from the experimental room walls. Participants were instructed to avoid the shots by moving around the experimental room and destroy the robots with their fingers. Finally, participants surfed the Internet with Microsoft Edge (Microsoft Corporation, Redmond, WA, USA). The experimenter instructed the participants to find a specific video or as much information as they could about a specific word. If necessary, the experimenter could help participants with the MRGlass device. Except for the Internet surfing task (with participants standing upright), participants had to move around the experimental room, sometimes using fast displacements. For example, during the Roboraid task, participants had to move fast or to do some fast knee or hip flexions to avoid laser shots.

At the end of the MRGlass session, participants responded to a survey to determine subjective feeling of visual fatigue, pain (location and intensity), comfort, autonomy, loss of balance, notions of time and reality and possibility to be used in working situations.

### Tasks—control condition

The study design was the same than during the MRGlass session, excepting that the participants did not wear the MR device, nor respond to the specific survey. Tasks during the control condition replicated the MRGlass tasks but without any device or using a tablet (iPad, Apple Inc., Cupertino, CA, USA). Accordingly, movements or displacements around the experimental room were also requested. Tasks—(i) Jenga, (ii) Photo Measures, (iii) Manual task, (iv) “Tennis-ball raid,” and (v) Internet surfing—were presented in the same order as in MRGlass condition. Briefly, Jenga (NaturalMotion Games Ltd, Oxford, UK) replicated Tower block on the tablet. Photo Measures was achieved using the following tablet application (Photo Measures; Big Blue Pixel Inc., Montreal, Canada). The manual task was the same as in MRGlass but with a paper manual. “Tennis-ball raid” replicated Roboraid but in “real life.” An experimenter, placed four m in front of the participants, played the role of the robots and threw tennis balls at the participants. Participants were instructed to avoid or catch the balls (each 50% of the ball throws). When the experimenter indicated the participant to catch the ball, the ball could be thrown in different directions (up, down, left, right) but with reasonable distances (maximum one step far from the participants). Balls were thrown at approximately 0.2 Hz frequency to replicate Roboraid application as close as possible. Weak throws were performed in order to be a feasible task and to avoid any anxiety related to a potential ball hit. Internet surfing was the same as in MRGlass but performed with the tablet (Safari, Apple Inc., Cupertino, CA, USA). During the Jenga and Internet surfing task, participants stood upright.

### Function tests

Function tests (baseline, post#1, post#2, post#3) began by asking participants to report their feeling of general fatigue (here called rate of perceived exertion) using a standard visual analog scale (the left hand end of the scale was referred as no fatigue = 0 and the right end as extreme physical and intellectual fatigue = 10) and any discomfort with the MR glasses (location of discomfort and intensity: very mild, mild, moderate, intense, very intense). Measurements were then made in a fixed order for the following tests: rate of perceived exertion; bipodal balance; precision task; unipodal balance; five words memory; Stroop test.

Bipodal and unipodal balance tests were performed on a posture platform (Posture Win, Techno Concept, Cereste, France) without shoes. During the bipodal balance test, feet were aligned with the drawings on the platform (10° angle between feet and three cm distance between the medial aspect of the heels). Participants stood upright with legs extended. They were instructed to keep their hands along the trunk and look at a mark on the wall (160 cm high and 250 cm away). Participants had to maintain this position for 51.2 s. During the unipodal balance test, participants stood upright on the right foot with the right leg extended. The right foot was positioned in a standardized position (heel-first toe axis aligned with the center of the platform). The left leg was flexed with a 90° knee angle, the left knee maintained in contact with the right knee. Participants were instructed to hold their hands-on hips and look at the same mark on the wall. This position was maintained for 25.6 s. In case of error (incorrect position or left foot touching the platform) the test was repeated.

The precision task test started on the posture platform with the same standing position used for the bipodal balance test. A filled glass of water was placed on a table in front of the participant in a precise position (floor-table surface distance = 50 cm; participant-glass distance = 60 cm). Participants were instructed to take the glass off the table, bring it to their mouth and then put it back on the table in the same position. They were requested to accomplish this task in 5 s. The task was repeated five times with a 4 s rest between repetitions. The means of the five tests are reported.

The memory test used a predefined list of five words that were presented to participants at the very beginning of the function tests in a standard fashion (Mormont, Jamart & Robaye, 2012). Participants were asked to read the list of words then to repeat each word eyes closed and to give the word associated with the experimenter’s clue. A clue was predefined for each word, for example, for banana “tell me the name of the fruit.” Then, for the memory test itself, participants were asked to recall the list of five words. When a word was forgotten, the experimenter could give the clue. Participants could take 20 s to recall a forgotten word before the clue was given; 40 s was allowed for full completion of the memory test. Results are expressed in number of forgotten words and number of clues given.

The color-word Stroop test (Stroop, 1935) was performed on a laptop using commercially available software (Unity Player, Unity Technologies, San Francisco, CA, USA, http://www.unity3d.com). Briefly, participants were to name the color of the ink in which a color name was presented with a possible conflict between the printed color name and the ink color (e.g., the word green written in blue letters). For that, words of four possible colors (red, blue, green, yellow) appeared centrally on a black background. Participants were given 60 s to select as quickly as possible the color of the word with the computer mouse. Results are expressed as total number of responses and percentage of errors.

### MR device and sensors

A HoloLens MR device (Microsoft Corporation, Redmond, WA, USA) running on Windows 10 was used during the MRGlass session. The head-mounted MR device had a total mass 579 g and has previously been tested in several conditions, for example, surgery or while visualizing complex structures (Hoffman & Provance, 2017; Qian et al., 2017; Tepper et al., 2017; Hanna et al., 2018). Care was taken to optimally position the MR device on the participant’s head to ensure comfortable movement. For this specific MR device, the head acts as a pointer. Arms and fingers, placed semi-flexed in front of the head/device are used as a controller to point, select, commutate, or exit applications with different movements according to the command required.

Bipodal/unipodal balance and precision tests were performed on a posture platform (Posture Win, Techno Concept, Cereste, France). During all balance tests, the position of the center of pressure was measured in mediolateral and anteroposterior directions at a 40 Hz sampling frequency. The surface area of the center of pressure and the velocity of the center of pressure displacement were retained for analysis (Paillard & Noé, 2015). The velocity was synchronized with EMG and the mean root mean square amplitudes were calculated.

Surface EMG was recorded using six pairs of silver-chloride electrodes. Electrodes were positioned in parallel with muscle fibers over the belly of the trapezius (TRA), deltoid (DEL), long head of biceps brachii (BB), latissimus dorsi (LAT), tibialis anterior (TA), and soleus (SOL) muscles. The center-to-center inter-electrode distance was two cm. Low impedance of the skin-electrode interface (<5,000 Ω) was obtained by shaving, abrading, and cleansing the skin. Electromyographic signals were registered using a wireless system (Aurion, Milan, Italy) connected to a Biopac system (Biopac, Santa Barbara, CA, USA). Signals were amplified with a bandwidth frequency ranging from 10 to 2,000 Hz (gain = 1,000). Root mean square values were calculated using 250 ms windows with 50% overlap and averaged to obtain a mean value during bipodal balance, unipodal balance, precision test, and also during the manual task within each 30 min series.

Heart rate was continuously registered during the whole experimental session using a portable heart rate monitor (Polar M400; Polar Electro Oy, Kempele, Finland). The mean heart rate during test sessions was calculated. Also, the mean heart rate was calculated during the 6 min of a specific task (Tower Block in MRGlass or Jenga in normal conditions) within each 30 min series.

### Statistical analysis

Mean values and standard deviation are presented. After checking application conditions using the Levene and Kolmogorov–Smirnov test, two-way analyses of variances with repeated measures were used (condition × time). Condition (MRGlass vs. Control) was the independent variable. Time was the repeated variable and corresponded to the four test time points. *F* ratios were considered significant at a *P*-level <0.05. When significant effects or interactions were present, a Student Newman–Keuls post-hoc test was performed. Furthermore, to assess the magnitudes of changes between conditions, effect sizes were determined using partial eta squared (ρη^2^). Values of 0.01, 0.06, and above 0.14 were considered to represent small, medium and large differences, respectively (Cohen, 2013).

## Results

### Rate of perceived exertion and discomfort

A significant main time effect was obtained for rate of perceived exertion (*F*[3,66] = 2.82, *P* < 0.05, ρη^2^ = 0.11). It increased significantly only after the first 30-min series (post#1) as compared to baseline (Table 1). No significant condition effect was obtained (*F*[1,22] = 1.62, *P* = 0.21, ρη^2^ = 0.06). Mean values during the whole procedure were 2.7 ± 1.6 and 2.1 ± 1.1 (of a 10-point scale) for MRGlass and Control, respectively. After the first 30-min series of MRGlass, five participants reported very mild discomfort affecting the head (*n* = 4) and eyes (*n* = 1) (Table 2). At the end of the three series with MRGlass, seven participants reported very mild discomfort affecting the head (*n* = 5), eyes (*n* = 1), and neck (*n* = 1). Immediately after the MRGlass session, four participants reported some pain over the head/neck, visual fatigue (*n* = 1), discomfort (*n* = 2), dependency (*n* = 2), or loss of balance (*n* = 0) (Table 2). In contrast, more mitigating results were obtained when considering the loss of reality (*n* = 5) and the loss of the notion of time (*n* = 7). Nine participants considered that MRGlass could be used by workers. Finally, no fall was observed during the whole experiment.

**Table 1:** Rate of perceived exertion, errors during the Stroop test, and average heart rate during the different test sessions and experimental series.

	MRGlass	Control
	Rate of perceived exertion	
Baseline	2.16 ± 0.39	1.75 ± 0.39
Post#1	2.75 ± 0.46	2.41 ± 0.46
Post#2	3.00 ± 0.44	1.91 ± 0.44
Post#3	2.83 ± 0.35	2.08 ± 0.35
	Number of errors during the Stroop test	
Baseline	0.91 ± 0.35	0.72 ± 0.35
Post#1	0.91 ± 0.29	1.18 ± 0.29
Post#2	0.82 ± 0.28	0.82 ± 0.28
Post#3	1.27 ± 0.37	1.09 ± 0.37
	Heart rate during tests	
Baseline	83.0 ± 2.9	83.4 ± 2.9
Post#1	82.4 ± 2.2	83.6 ± 2.2
Post#2	80.4 ± 2.5	81.1 ± 2.5
Post#3	78.2 ± 2.6	83.6 ± 2.6
	Heart rate during experimental series	
Series#1*	81.9 ± 2.5	85.6 ± 2.5
Series#2	78.8 ± 2.6	82.7 ± 2.6
Series#3	78.1 ± 2.5	81.9 ± 2.5

**Notes:**

Values are means ± standard deviation. MRGlass: condition with mixed reality glasses. A significant time effect was only observed for heart rate during experimental series with significantly greater values during the first series as compared to the two others.

* *P* < 0.05.

**Table 2:** Discomfort, fatigue, autonomy, reality, and notion of time using MRGlass (mixed reality glasses).

Queries	Answers
Did you feel any pain while wearing MRGlass?	Head/neck: Medium = 4, no = 8 Back: no = 12 Legs: no = 12
Did you feel any pain after wearing MRGlass?	Head/neck: Medium = 2, no = 10 Back: no = 12 Legs: no = 12
Did you feel any visual fatigue while wearing MRGlass?	Yes = 0, Normal = 4, No = 8
Did you feel any visual fatigue after wearing MRGlass?	Yes = 1, Normal = 1, No = 10
Are MRGlass comfortable?	Yes = 10, No = 2
Did you feel dependent or autonomous?	Autonomous = 10, Dependent = 2
Do you think MRGlass can be use in working situation?	Yes = 9, No = 3
Did you lose balance with MRGlass?	Yes = 0, No = 12
Did you lose your real environment with MRGlass?	Yes = 4, No = 8
Did you lose the notion of time with MRGlass?	Yes = 7, No = 5

**Note:**

Values are number of responses.

### Memory and Stroop test

During the five-word memory test, very few errors were noticed (range of error and clues given during each test session range [0;1]). No significant main effect or interaction was obtained for Stroop test performance (*F*[3,60] = 0.775, *P* = 0.51, ρη^2^ = 0.03 and *F*[3,60] = 0.22, *P* = 0.82, ρη^2^ = 0.01 for interaction in the total number of responses and the number of errors, respectively). For instance, percentage errors were 1.4 ± 1.6% and 1.5 ± 1.8% in MRGlass and Control conditions, respectively (Table 1) (total number of responses ranging from 47 to 58).

### Heart rate

Mean heart rate measured during test sessions and during the experimental series (Table 1) did not demonstrate any significant interaction (*F*[3,60] = 2.38, *P* = 0.08, ρη^2^ = 0.10 and *F*[2,40] = 0.01, *P* = 0.98, ρη^2^ = 0.01, respectively). However, mean heart rate measured during the experimental series showed a significant main time effect (*F*[2,40] = 20.8, *P* < 0.05, ρη^2^ = 0.51). It significantly decreased with time (83.7 ± 1.8, 80.7 ± 1.8, and 80.0 ± 1.8, during the first, second, and third series, respectively).

### Balance and EMG

**Figure 2:**
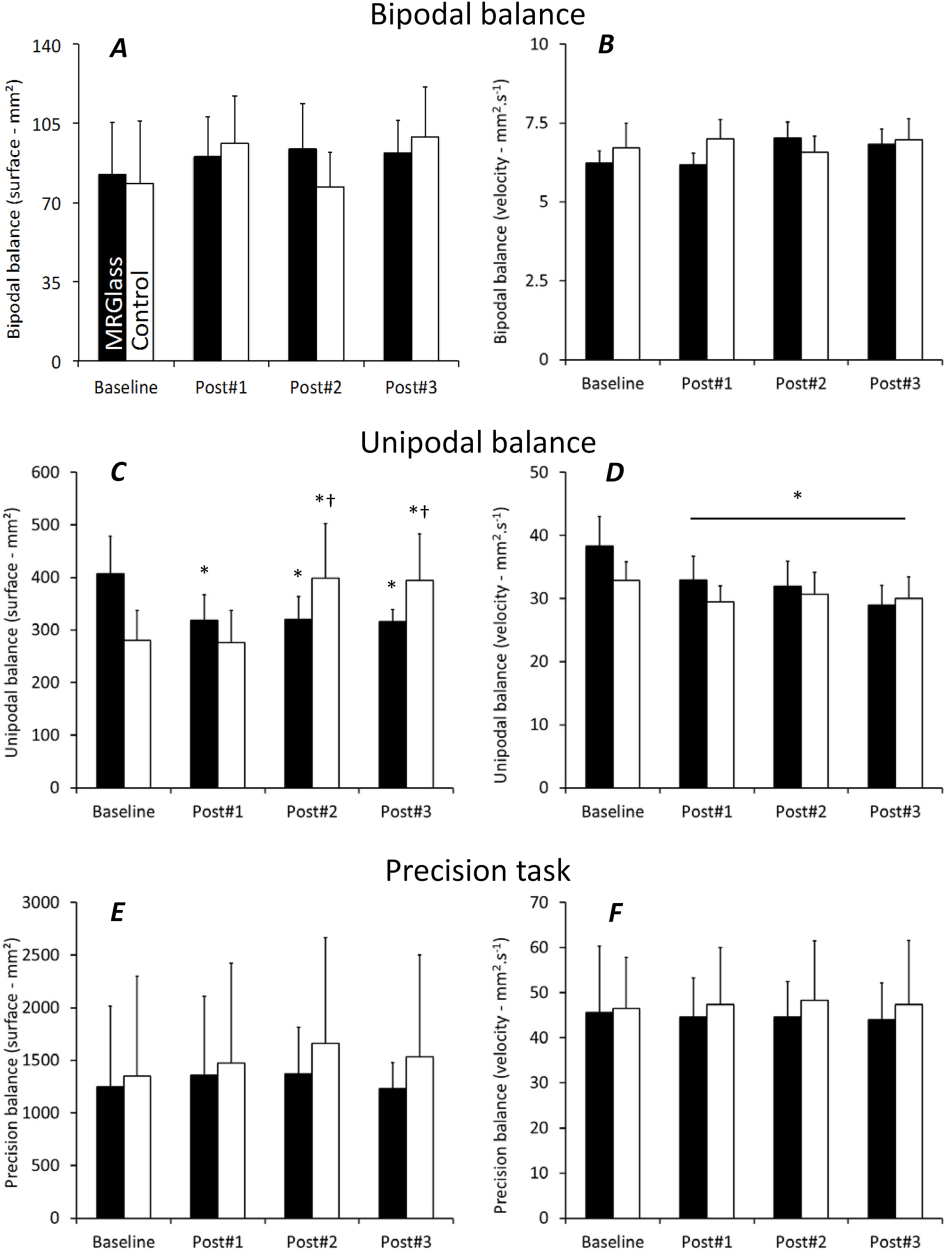
Centre of pressure surface area (A, C, and E) and velocity (B, D, and F) during the bipodal, unipodal, and precision balance tests. Black bars corresponded to MRGlass condition and white bars corresponded to Control condition. *, significant differences with baseline (*P* < 0.05); †, significant differences with Post#1 (*P* < 0.05). Values are means ± standard deviation.

During the bipodal balance test, no significant main effect or interaction were obtained for the center of pressure surface and velocity (*F*[3,66] = 0.45, *P* = 0.71, ρη^2^ = 0.02 and *F*[3,66] = 2.40, *P* = 0.07, ρη^2^ = 0.09, respectively) (Fig. 2). No significant main effect or interaction was observed for the EMG activity of the six muscles under investigation.

During the unipodal balance test, a significant interaction was obtained for the center of pressure surface (*F*[3,66] = 5.42, *P* < 0.05, ρη^2^ = 0.20). With time, surface area significantly decreased in MRGlass condition, while it significantly increased in Control condition (Fig. 2). For the velocity, only a significant main time effect was obtained (*F*[3,66] = 7.53, *P* < 0.05, ρη^2^ = 0.25). The mean velocity was significantly faster at baseline than during all three post-tests (Fig. 2). No significant main effect or interaction was observed for the EMG activity of the six muscles under investigation.

Finally, during the precision task, no significant main effect, or interaction was obtained for the center of pressure surface and velocity (*F*[3,66] = 0.39, *P* = 0.75, ρη^2^ = 0.02 and *F*[3,66] = 0.46, *P* = 0.70, ρη^2^ = 0.02, respectively). Significant interactions were obtained for the EMG activity of TRA, DEL, BB, and TA muscles (*P* < 0.05). For these four muscles, post-hoc analyses only revealed greater values at baseline in Control conditions as compared to post#2 and post#3 (*P* < 0.05). No other differences were obtained (neither between conditions nor for the other muscles).

### EMG activity during a specific task

**Figure 3:**
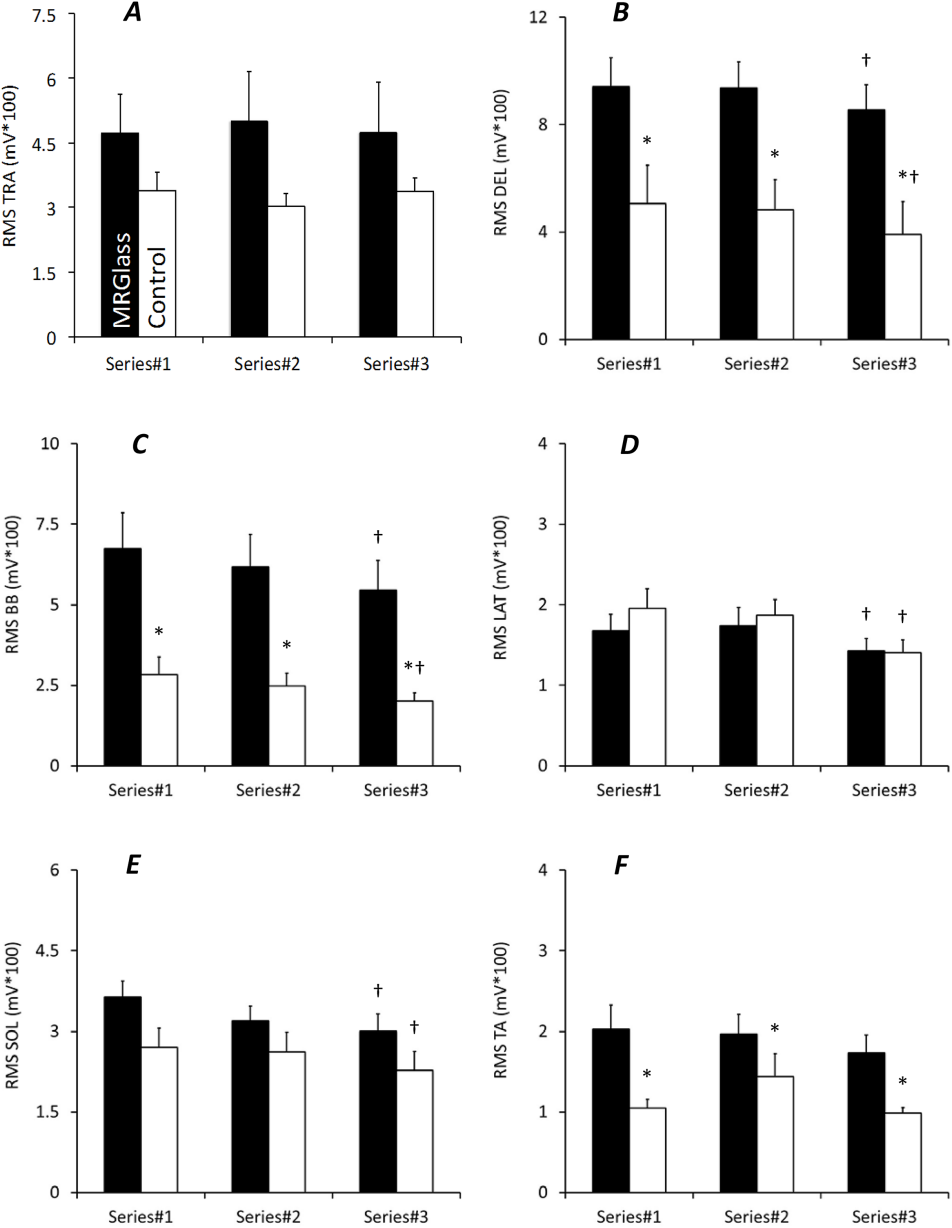
EMG activity measured during a specific task with MRGlass (black bars) and in Control condition (white bars). EMG activities are for trapezius (A), deltoïdus (B), long head of biceps brachii (C), latissimus dorsi (D), soleus (E), and tibialis anterior (F) muscles. *, significant differences with MRGlass (*P* < 0.05); †, significant differences with Series#1 and Series#2 (*P* < 0.05). Values are means ± standard deviation.

EMG activity was also measured during one task of each series (Fig. 3). No significant interaction was obtained for all six muscles. There was a significant main time effect for DEL, BB, LAT, and SOL (*P* < 0.05). For these muscles, post-hoc analyses revealed significantly lower values during the third series as compared to the first and second series (*P* < 0.05). A significant main condition effect was obtained for DEL, BB, and TA muscle (*P* < 0.05). For these three muscles, a significantly greater EMG activity was obtained in MRGlass as compared to Control condition (*P* < 0.05).

## Discussion

The aim of the present study was to investigate the effects of wearing an MR Hololens device on certain cognitive and physiological functions so as to determine its safety. Our results show that during specific tasks, wearing MR glasses affected unipodal balance and muscular activity minimally. No effects were registered for (i) cardiovascular function, (ii) bipodal balance and precision tasks, and (iii) cognitive function. One third of the participants noticed mild discomfort attributable to the headset fixation. Optimal positioning is required. As hypothesized, we can conclude that Hololens MR glasses appeared to be safe and can be used in working environments without any risk of fall on flat surfaces.

It could be speculated that tasks performed while wearing VR or AR devices would be more demanding. For example, Baumeister et al. (2017) recently indicated that head-mounted devices can increase cognitive load. Here, we tested this affirmation considering three different domains: mental, cardiovascular, and neuromuscular behavior. Compared with the results of similar tasks performed without any MR device, the results obtained with MR glasses demonstrated no alteration of memory, attention, or energy expenditure and only slight differences in muscle activation. These observations are in discordance with certain findings reported in previous studies. Some authors have registered enhanced attention (Ferrari et al., 2014) or increased heart rate (Nalivaiko et al., 2015) in VR environments. In contrast, others showed that VR eyeglasses successfully decreased pain perception and anxiety (Asl Aminabadi et al., 2012) or pulse rate (Sullivan et al., 2000) during dental treatments. These differences could likely be attributed to the different systems under investigation and to potential differences between VR and MR.

Most of the differences we observed between test and control conditions were related to the amplitude of muscle activation. Except for slight time-related differences, which could be attributed to learning effects, greater activation amplitude was observed in DEL, BB, LAT, and SOL muscles in the MR condition. Previously, only one study has tested the effects of AR on muscle activation in healthy individuals (Moon et al., 2015). Although the authors reported increased contractile activity contributing to greater muscle fatigue, they only measured activity of lower-limb muscles involved in the ski simulation under study. The specificity of this task would exclude comparisons with our results. In our study, the greater muscle activations observed in upper-limb muscles were not surprising. Indeed, with this specific device, the head acts as a pointer and the upper-limb is used as a controller (selection, application, commutation…). These actions produce a twofold increase in EMG activity to maintain the semi-flexed upper-limb in front of the individual’s head. Interestingly, perceived fatigue or discomfort was not associated with this increased activity level.

Moreover, despite the weight of the device (579 g), wearing MR glasses did not lead to a significant increase in the level of neck muscles activity since no increase in TRA activity was recorded. There was, however, an increase in the contractile activity of the SOL, a mostly slow-postural muscle. Enhanced SOL activity would stiffen the lower-limb, allowing less movement around joints exposed to possible disturbance when wearing MR glasses. Such increased stiffness has previously been observed in unstable situations (Horak & Nashner, 1986). But the results of the balance tests, as well as the subjective feeling of balance discussed below, are in contradiction with this instability argumentation. Further measurements are needed to clarify this point using different balance tests and more particularly using stable and/or unstable conditions.

Fall prevention and safety of use are of paramount importance to limit injuries, and more particularly traumatic brain injuries (Paci, Infante-Rivard & Marcoux, 2017). Immersion into virtual environments could be considered as a risk factor. For example, various accidents have been associated with AR environments such as with the recent *Pokémon Go* game. Collisions, accidents and increased exposure to mosquito-borne diseases have been reported (Oidtman et al., 2016). Safety of use during the more controlled situations in MR environments not using screens tested here has been validated since wearing MR glasses had no effect. No alteration of balance was observed during bipodal or unipodal tests. Moreover, the muscle activation strategies applied to control balance were similar between MRGlass and Control conditions.

For unipodal balance, we recorded a smaller center of pressure surface area in MRGlass than in Control conditions. This different behavior can be attributed, at least in part, to the fact that wearing MR glasses slightly reduces the visual field available for focusing on a target. Others have previously demonstrated that vision becomes preponderant to maintain balance when the visual field is reduced laterally (Assaiante, Marchand & Amblard, 1989). None of our participants reported any loss in balance while wearing the MR glasses, but further work will be needed to test balance in different ecological conditions and more particularly in unstable situations in order to confirm this subjective feeling. Similarly, our precision task had no effect on balance. Together with the results of other balance tests, this finding indicates that these specific MR glasses can be used safely, with no increase in the risk of falls in stable flat conditions. The beneficial effect that long-term application of AR has on balance has already been demonstrated in various populations such as older adults (Mirelman et al., 2016; Dockx et al., 2017). Moreover, training with AR can limit the risk of falls via enhanced confidence or habit-related learning effects in hazardous working situations (Hsiao et al., 2005).

One important issue for using VR/AR/MR is related to individuals defined as responders or non-responders. If used in learning or working environments, glasses should be comfortable without any associated pain or fatigue. Although a few participants (one-third) reported mild pain located at the head when wearing or after wearing the MR glasses, in general participants did not mention any additional fatigue, whether global or visual. This mild discomfort is discordant with a previous study (Gamberini et al., 2015) where the authors were unable to register any discomfort among participants wearing a 240 g device during 5-min tasks. The mild discomfort registered in our study might be related to the weight of the head-mounted device (579 g) and the duration of the tasks (90 min). However, the difference between pain sensations reported when wearing and after wearing MR glasses revealed that the discomfort was partly mechanical in nature. The device has to be mounted on the head tight enough to allow a wide range of movements. Inadequate positioning or tightening would favor unwonted discomfort. According to this speculative observation, appropriate controlled setup conditions must be defined. In addition, a few participants indicated that the device limited their autonomy. These results point out the importance of an appropriate supervised setup and familiarization procedure. Moreover, because most individuals reported that the experience involved a disconnection from reality and a loss of the notion of time, we recommend using AR devices for specific purposes—for example, learning experiences or while working—under supervision and alternated with frequent rest periods. This would limit unsafe behavior. As previously reported (Ghasemi et al., 2017), training and supervision are the most important factors to limit unsafe behaviors. Finally, it is important to note that most of our participants felt that MR devices could be used in working situations, a finding similar to the conclusions of a previous study where the participants reported that the AR experience was a pleasant one (Gamberini et al., 2015). This could be an advantage to increase motivation during learning exercises and in some working situations (Trojan et al., 2014). Virtual environments should be carefully selected not to increase uselessly individuals’ anxiety levels (Hartanto et al., 2014).

The present study presents some limitations related to the device tested, the different tasks, and the function tests. Firstly, the present study was performed with a Hololens MR device. Conclusions are therefore only related to this specific head-mounted device. Secondly, specific tasks have been performed during the experiment. Tasks were selected so as to allow replication in a control condition. Some of these tasks (in the MRGlass or control condition) could have yield to different mental, physical demands or anxiety level that could consequently alter some of our measurements (e.g., heart rate or subjective ratings). More particularly, to mimic roboraid in the control condition, we used tennis balls threw by one experimenter to participants. This control condition (that used tennis balls) could have yield to greater discomfort or anxiety level that could have masked potential discomfort directly caused by MR glasses. Thirdly, the present study used simple and short tests in order to investigate the different cognitive and physiological functions. Additional or more complete measurements could have been performed to avoid any ceiling effects (e.g., during the five-words memory test). This study should therefore be replicated with other tasks, tests and with more volunteers in order to increase statistical power.

## Conclusion

This study is one of the first to test the use of Hololens head-mounted MR glasses in different intellectual and manual situations while exploring cognitive, cardiovascular, and neuromuscular functions. Our findings clearly indicate that the MR device under investigation is safe since it does not alter balance and does not produce additional cognitive and physical fatigue compared with the control condition. According to observations achieved during the whole experiment, some recommendations can be formulated: (i) participants should be receptive to such devices; (ii) glasses should be adequately positioned; (iii) familiarization should be efficient and supervised to favor autonomy; and (iv) when used in learning or working environments, supervision, and frequent breaks are mandatory to avoid unsafe behaviors.

## Supplemental Information

10.7717/peerj.5847/supp-1 Supplemental Information 1 Raw data for all conditions and all tests. Conditions are codes as MRglasses or Control. Tests are presented as separated excel sheets.

## Additional Information and Declarations

### Data Availability

The following information was supplied regarding data availability: The raw data are provided in a Supplemental File.
